# Acute Severe Hepatitis B Virus Infection in Previously Vaccinated Patient during Acalabrutinib Treatment

**DOI:** 10.3201/eid3202.251111

**Published:** 2026-02

**Authors:** Lukasz Rojek, Tomasz Wnuk-Lipinski, Iwona Marek, Hubert Grabowski, Krystian Adrych

**Affiliations:** Authors affiliation: Medical University of Gdansk, Gdansk, Poland

**Keywords:** Hepatitis, viruses, hepatitis B, hepatic insufficiency, liver transplantation, acalabrutinib, Poland

## Abstract

We describe acute severe hepatitis B virus (HBV) infection with liver failure requiring transplantation in a patient in Poland treated with acalabrutinib. The patient was fully vaccinated against HBV and had adequate HBV antibody titers and no HBV exposure documented before therapy. Differential diagnoses for jaundice should consider HBV in patients receiving acalabrutunib.

Hepatitis B virus (HBV) infection is the most prevalent long-term viral disease globally (*1*). The World Health Organization estimates that ≈254 million persons worldwide are chronic carriers of the hepatitis B surface antigen (HBsAg) (*2*). HBV is one of the most common causes of liver cirrhosis and hepatocellular carcinoma (*3*). Widespread hepatitis B (HepB) vaccination has been available since 1981 and remains the most effective strategy for preventing HBV infection (*1*,*4*). Immunocompetent adults and children with vaccine-induced HBsAg antibody levels >10 mIU/mL after a full >3-dose HepB series are generally considered seroprotected and classified as vaccine responders (*5*). Some reports suggest a loss of immunity against HBV, manifested by a decline in HBs antibody titers (*6*). That phenomenon is particularly observed in patients receiving B cell–depleting therapies, such as anti-CD20 monoclonal antibodies (*7*).

Bruton tyrosine kinase (BTK) is a vital protein involved in B cell proliferation, maturation, and differentiation (*8*). Its central role in the B-cell antigen signaling pathway has made BTK a key target for developing therapies to treat B-cell malignancies (*8*). Acalabrutinib, a second-generation BTK inhibitor, is approved for treating chronic lymphocytic leukemia (*8*). Patients with hematologic malignancies undergoing BTK inhibitor therapy can have an intermediate risk for HBV reactivation (*9*). We report possible primary acute hepatitis B with liver failure, despite successful prior vaccination against HBV, in a patient in Poland who was receiving acalabrutinib.

A 58-year-old female patient was referred to the hospital because her general condition had deteriorated, and she had jaundice, abdominal pain, nausea, and anorexia. She had symptoms of an upper respiratory infection 6–7 weeks earlier, which had resolved. The patient had been treated with oral acalabrutinib (100 mg 2×/d) for chronic lymphocytic leukemia since April 2023. Normalization of peripheral leukocytes and partial remission of nodal changes were achieved with treatment. However, in early February 2024, hematologist observed an increase in liver enzyme levels (Table 1) and discontinued acalabrutinib treatment.

**Table 1:** Blood test results from a case of severe HBV infection in previously vaccinated patient during acalabrutinib treatment*

Biochemical parameter	Before HBV infection	Day of hospitalization						
		−13	−3	0	1	2	3	4
Total bilirubin, mg/dL	0.84	2.19	23.04	43.02	32.17	33.76	33.89	35.66
Alanine aminotransferase, U/L	27	NA	2,688	3,780	2,603	2,024	1,340	875
Aspartate aminotransferase, U/L	29	945	3,877	4,400	3,146	2,384	1,058	NA
Alkaline phosphatase, U/L	NA	NA	212	211	164	NA	147	NA
Gamma-glutamyltransferase, U/L	NA	NA	421	233	171	NA	107	NA
Ammonia, µmol/L	NA	NA	NA	221	NA	NA	92	NA
International normalized ratio	NA	NA	2.89	>6.12	>6.12	>6.12	>8.6	>8.6
Creatinine, mg/dL	0.79	0.66	0.69	1.63	2.33	3.13	3.94	4.43
Sodium, mmol/L	141	136	136	129	126	121	118	117
Potassium, mmol/L	4.3	4.5	4.5	6.4	5.9	5.1	4.6	4.6
Hemoglobin, g/dL	13.7	13.9	NA	14.3	11.8	11.1	9.9	9.5
Erythrocytes, × 10^12^ cells/L	4.02	4.13	NA	4.4	3.55	3.38	3.0	2.9
Platelets, × 10^9^/L	207	150	NA	245	159	135	89	80
Total leukocytes, × 10^9^ cells/L	8.01	6.1	NA	8.33	7.43	6.24	5.27	5.04
C-reactive protein, mg/L	1.12	NA	21.17	14.85	9.38	NA	4.08	NA
Procalcitonin, ng/mL	NA	NA	NA	NA	0.84	0.81	NA	0.61

*HBV, hepatitis B virus; NA, not available.

The patient’s medical history included type 2 diabetes mellitus, hypothyroidism, and obesity (body mass index 36). Her surgical history included hysterectomy, bariatric surgery via adjustable gastric band, and cholecystectomy.

The patient was previously vaccinated against HBV with 3 HepB doses before 2019. In January 2023, her HBs antibody level before acalabrutunib initiation was 120.25 mIU/mL. Total hepatitis B core (HBc) antibodies were not detected.

At admission, the patient had severe jaundice (total bilirubin 43 mg/dL [reference range 0.1–1.2 mg/dL]), hepatomegaly, grade 1 ascites without peripheral edema, anuria, and mean arterial pressure 65 mm Hg (reference >60 mm Hg). She was afebrile, and her mental status was at baseline without evidence of encephalopathy.

Ultrasound examination showed the liver without cholestasis or focal changes and flow in the hepatic veins, portal veins, and hepatic artery within reference ranges. Ultrasound also revealed lymphadenopathy in the liver hilum and fluid in the peritoneal cavity.

Virology results were positive for HBsAg, hepatitis B envelope (HBe) antigen, HBc total antibodies, and HBc IgM. PCR on HBV DNA confirmed high viremia at 1.67 × 10^7^ IU/mL (Table 1; Table 2). Hepatitis C virus, Epstein-Barr virus, and cytomegalovirus infections were excluded.

**Table 2:** Virologic test results a case of severe HBV infection in previously vaccinated patient during acalabrutinib treatment*

Test	Before starting acalabrutinib		Acute hepatitis B†	After liver transplant‡
	2022	2023		
HBsAg	Undetectable	NA	Detectable	Undetectable
HBeAg	NA	NA	Detectable	NA
Anti-HBs, mIU/mL	104	120.25	0	41.8
Anti-HBc total antibodies	Undetectable	Undetectable	Detectable	Detectable
Anti-HBc IgM	NA	NA	Detectable	NA
HBV DNA, IU/mL	NA	NA	1.67 ×10^7^	Undetectable

*Anti-HBS, antibody to HBsAG; anti-HBc, antibody to HBcAG; HBcAg, hepatitis B core antigen; HBeAg, hepatitis B envelope antigen; HBsAg, hepatitis B surface antigen; HBV, hepatitis B virus; NA, not available. †February 2024. ‡September 2024.

Eventually, acute viral hepatitis B with multiorgan failure involving the liver, kidneys, circulation, and coagulation was diagnosed. Entecavir was initiated (0.5 mg orally 1×/d), and empirical antibiotic therapy (750 mg intravenous cefuroxime 2×/d) was introduced. Because of high ammonia in blood serum (221 µmol/L [reference range 18–72 µmol/L]), we also administered oral rifaximin (400 mg 3×/d), intravenous L-ornithine L-aspartate (50 g total dose), and oral lactulose (20 mL 3×/d). Because hepatorenal syndrome was causing acute kidney injury, we administered 20% albumin (100 mL intravenously 2×/d) and continuous intravenous infusion of terlipressin (2 mg/d). Because renal function worsened and fluid overload increased, we implemented hemodialysis and supported circulation by a noradrenaline infusion. Despite treatment, the patient’s condition deteriorated, and she was transferred to the transplant department, where successful liver transplantation was performed 19 days after onset of jaundice.

Current guidelines emphasize the need for comprehensive HBV serologic screening before initiating immunosuppressive therapy, including testing for HBsAg and HBc and HBs antibodies (*10*). However, those guidelines do not provide clear recommendations regarding monitoring HBs antibody levels during treatment. Routine HBs antibody monitoring might have prevented complete loss of vaccine-induced immunity and subsequent severe clinical course in this patient.

This report highlights that screening for HBV hepatitis is vital for patients treated with acalabrutunib, even those appropriately vaccinated against hepatitis B. HBV reactivation has been reported in patients who had previous HBV contact and initial HBc antibodies (*9*). Our patient’s history of BTK inhibitor treatment shows that differential diagnosis of jaundice or infection should also consider diseases for which patients have been vaccinated.
